# Cervical cancer management in Zimbabwe (2019–2020)

**DOI:** 10.1371/journal.pone.0274884

**Published:** 2022-09-21

**Authors:** Petmore Zibako, Nomsa Tsikai, Sarah Manyame, Themba G. Ginindza

**Affiliations:** 1 Discipline of Public Health Medicine, School of Nursing and Public Health, University of KwaZulu-Natal, Durban, South Africa; 2 College of Health Sciences, University of Zimbabwe, Harare, Zimbabwe; 3 Cancer & Infectious Diseases Epidemiology Research Unit (CIDERU), College of Health Sciences, University of KwaZulu-Natal, Durban, South Africa; University of Brescia: Universita degli Studi di Brescia, ITALY

## Abstract

**Background:**

Globally, cervical cancer is the fourth most commonly diagnosed cancer amongst women, and it is especially common in low- and middle-income countries (LMICs). The aim of the study was to determine the current patterns and characteristics of CC management in Zimbabwe in the HIV pandemic era, including the knowledge, attitude and practice of patience.

**Methods:**

The study was a mixed method which incorporated a cross-sectional survey of 408 CC patients which was conducted from October 2019 to September 2020 using an interviewer administered paper questionnaire. The study was conducted at Parirenyatwa hospital, the only cancer treating public health facility in Harare, Zimbabwe. Differences in study outcome by categorical variables were assessed using the Person Chi-square (χ^2^) test. Odds ratios (unadjusted and adjusted) and 95%CIs for potential risk factors associated with the outcome were estimated using logistic regression model.

**Results:**

From a total of 408 CC patients recruited into the study no prevention mechanism was available or known to these patients and only 13% knew that CC is caused by Human papillomavirus. Only 87 (21%) had ever been screened for CC and 83 (97%) of those who had been screened had the visual inspection with acetic acid procedure done. Prevention (screening uptake) is statistically high among the educated (with secondary education OR = 9.497, 95%CI: 2.349–38.390; with tertiary OR = 59.381, 95%CI: 11.937–295.380). Late presentation varied statistically significantly with marital status (high among the divorced, OR = 2.866; 95% CI: 1.549–5.305 and widowed OR = 1.997; 95% CI: 1.112–3.587), was low among the educated (Tertiary OR = .393; 95% CI: .166-.934), low among those living in the rural (OR = .613; 95% CI: .375-.987), high among those with higher parity OR = 1.294; 95% CI: 1.163–1.439). Less than 1% of the patients had surgery done as a means of treatment. Radiotherapy was administered to 350 (86%) of the patients compared to chemotherapy administered to 155 (38%). A total of 350 (86%) have failed to take medication due to its unavailability, while 344 (85%) missed taking medication due to unaffordability. Complementary and alternative medicines were utilized by 235 (58%). Majority, 278 (68%) were HIV positive, mainly pronounced within age (36–49 years OR = 12.673; 95% CI: 2.110–76.137), among those with higher education (secondary education OR = 4.981; 95%CI: 1.394–17.802 and in those with no co-morbidities (893.956; 95%CI: 129.611–6165.810).

**Conclusion:**

CC management was inadequate from prevention, screening, diagnosis, treatment and palliative care hence there is need to improve CC management in Zimbabwe if morbidity and mortality are to be reduced to acceptable levels. Education helped improve prevention, but reduces chances of diagnosis, working as a doubled edged sword in CC management Prevention was high among the educated. Those in rural areas experience poor CC management. It should be noted that general education is good; however it must be complimented by CC awareness to improve CC management outcomes holistically. Cervical cancer management services need to be decentralized so that those in rural areas have easy access. Given that those with co-morbidities and high parity have better CC management, CC services need to be tied to co-morbidity and antenatal/post-natal care and management services.

## Introduction and background

There are different levels of cervical cancer (CC) management process, starting from prevention, screening, diagnosis, treatment and palliative care [1]. The availability of prevention, screening, diagnosis, treatment and palliative care services is inversely proportional to the economic status of a country [1]. CC is a threat to women’s health as globally there were 570 000 new cases and 311 000 deaths in 2018 [2]; and 85% of these cancer cases occur in low-middle income countries (LMICs) [2]. Cervical intra-epithelial neoplasia (CIN) takes 10-20yrs before it becomes invasive CC [3], if not treated, therefore window of opportunity to screen, detect and treat to avoid invasive CC is there [4]. Availability of excellent screening services in high income countries(HICs) like United States of America (USA) and United Kingdom (UK) has provided for their lowest morbidity and mortality of CC [5].

CC is a preventable and curable disease; preventable by vaccination and screening and curable if identified at an early enough stage [6], implying stage of presentation is also very critical in CC management. It is gradually becoming a rare disease in many high-income countries (HICs) [4]; this is not the case in most LMICs [7]. Cancer alongside other major non-communicable diseases (NCDs), such as cardiovascular diseases, diabetes and chronic respiratory diseases cause over 60% of total global mortality every year [8]. Global CC incidence range from 1 to 75 per 100 000 women [2] The worst part is that CC still has less recognition as a major public health issue compared to traditional ailments such as HIV and AIDS, malaria and tuberculosis (TB), mostly in LICs [3]. This is despite the reality that today cancer is killing more people than HIV and AIDS, TB and malaria combined; 7.6 million people are dying from the disease globally, every year [3]. Additionally, each year 14 million are diagnosed of cancer and the figure is projected to be above 21million by 2030 if nothing is done [9]. Cervical cancer is one of AIDS-defining diseases and the most common AIDS-related cancer in women with most CC patients generally being HIV positive [10]. HIV prevalence was 12.9% (1.23 million adults) in Zimbabwe as of 2020, the HIV prevalence was higher among women than men (15.3% against 10.2%) and approximately ninety percent of those who were on antiretroviral drugs were virally suppressed [11]. Eighty-seven percent of adults who were living with HIV were aware of their status and ninety-seven percent of those who were aware of their status were on antiretroviral drugs [11].

Treatment options for CC patients include radical hysterectomy, external-beam radiation, brachytherapy and chemotherapy. CC patients in sub-Saharan Africa (SSA) present with advanced disease despite the fact that, in 2010 radiotherapy treatment facilities were available in 23 of 52 African countries, brachytherapy was available in 20 countries and only five African cancer registries were included in the World Health Organization publication (WHO) [12]. African countries share about 85% of morbidity and 87% of death due to CC [12]. The average incidence of CC in SSA is 35 per 100,000 women, and 23 death occur per 100,000 women every year [13]. WHO in 2012 estimated that 3.65 billion United States dollars is required to combat CC in LMICs for vaccination of girls, screening services and treatment [4]. Screening and treatment remain critical components of CC control over the coming generation even if the most effective vaccination program is put in place, because of millions of women already infected with human pappiloma virus (HPV). CC is a disease that demonstrates inequities of access to health care resources [14]. Cervical screening is the process of detecting abnormal tissue or cells in the cervix before CC develops [15]. The frequency of screening is determined by method of screening used; every three years with Pap smear, Pap smears with HPV testing: five years [15]. This study was considering screening as those that have done it once in their lifetime.

In 2012, 6 LMICs had national immunization programs for HPV vaccination and a minority of women are screened for CC in their lifetime in LMICs [4]. In 2013, 90% of 236,000 women who died from CC were from developing nations [16]; representing a failure to implement a functional CC control strategy. The ways to prevent and treat CC are well known, death from CC is considered preventable and unnecessary.

CC is the most commonly diagnosed cancer among women in Zimbabwe, 2,270 cases and 1,451 deaths reported in 2014 [17]. The highest frequently occurring cancer among female Zimbabweans of all races in 2014 was CC (19.8%) [17]. There were 2,270 new CC cases in 2014 in Zimbabwe translating to 34.5 per 100 000 women per year, majority (over 80%) of whom are residing or treated in the capital city, Harare [18]. According to GLOBCAN2018 Zimbabwe is among countries with incidence greater than 40 per 100 000 women. In Zimbabwe CC mortality rate is 64% [19]. These figures could be lower than the actual figures because many cases go unrecorded in rural areas. CC treatment is highly centralised in Zimbabwe with only two public centres offering comprehensive services, that is, Parirenyatwa Group of Hospitals in Harare and Mpilo Hospital in Bulawayo, which are the two largest cities.

The aim of this research was to establish the current status of cervical cancer management in Zimbabwe and the specific objectives were to establish patterns and characteristics of cervical cancer management in Zimbabwe as well as to determine the prevalence of HIV among CC patients in Zimbabwe. The study hypothesized that there is room for improvement in CC management in Zimbabwe.

## Material and methods

### Study settings, study design and study population

The study was conducted at Parirenyatwa group of Hospitals (radiotherapy, outpatients’ oncology clinic, gynaecology wards and a female ward for cancer patients). Parirenyatwa hospital is the only cancer treating public health facility in Harare where CC patient are treated. Treatment for: stage 1ab include surgery and internal radiation for women who cannot have surgery, stage 2a include surgery, radiotherapy and chemotherapy, stage 2b include surgery, radiotherapy and chemotherapy, stage 3abc include radiotherapy with or without chemotherapy and stage 4ab include radiotherapy with or without chemotherapy. The study applied mixed-methods approach incorporating a cross sectional survey using a semi-structured questionnaire (see S1 File). A questionnaire with a combination of closed ended questions (to quantify responses) and open ended to explain in detail the experiences was used. The data was both quantitative and qualitative collected and processed in a concurrent/embedded manner. Triangulation of methodologies, data sources, data collection techniques helped to ensure robust results.

Participants for the study included CC patients who were coming for treatment at Parirenyatwa Group of Hospitals at radiotherapy centre, oncology clinic in the outpatients department, gynaecology wards as well as CC patients who were admitted in the female cancer ward from October 2019 to September 2020. No categorisation of patients was done given the nature of recruitment utilised- any characteristics were solicited as part of the study and was not know a-prior or ex ante, but became useful in ex post as analysis unfolded which prevented bias towards pre-conceived outcomes. The grouping that emerged were then utilised to understand the key outcomes: CC management components (prevention, diagnosis, treatment and care).

### Sample size and sampling technique

Dobson’s sample size calculation formula was used to determine the minimum sample size (384) with the assumption that the outcomes being evaluated response distribution is 50% and the desired confidence level is 95%. To cater for incomplete/unusable questionnaires 420 were administered and 408 were complete and usable. to meet the objectives of the study: how cancer is being managed in Zimbabwe as well as the prevalence of HIV among CC patients.

A purposive sampling technique of patients with histologically proven CC was used to select CC patients. Cancer patients who came for routine check-ups, treatment and those that were admitted at Parirenyatwa hospital were enrolled as they became available at the hospital until the required sample size was reached. The enrolment was done every day.

### Recruitment

CC patients were approached to participate in the survey through a purposive sampling approach, those who were willing and available to respond to the questions (willingness and availability confirmed by signing a written informed consent). A numbering system was used for each questionnaire. Interviews were conducted by the principal investigator and one research assistant who was an oncology nurse.

Cervical cancer patients who came to Parirenyatwa hospital during the data collection period that consented to take part in the study were included. Very ill patients were not included for example those who were not able to speak or bed ridden as well as those below the age of 18 to accommodate only those who can legally consent.

### Data management and analysis

Interviewer-administered paper questionnaire was used for data collection on patients. The instrument administer would read the question, get a response from the respondent (CC patient who have consented) and write/ tick the response on the printed questionnaire. To test the instrument, a pilot study of 20 patients was done and necessary changes were made to the questionnaire. The interviews were done during the morning up to 12 mid–day with responses captured on the response sheet. Where clarity or additional detail was needed, probing techniques were used.

Data was captured in electronic format using Excel in preparation for uploading into the software for analysis. The electronic data was backed up on different devices and physical questionnaires were stored in a lockable cupboard with no information linking respondents to the data. Anonymity was ensured in the study by not asking for names of respondents and participants, but codes were used.

A pilot study helped verify effectiveness of instrument; training of research assistants was prioritised and double-checking during data entry. Validity and reliability test were done prior to full-scale survey and data analysis. Piloting of the study questionnaire was done to increase the internal reliability of the questionnaire. To ensure validity, responses were double-checked with respondent and confirmatory factor analysis was used to measure validity objectively. In addition, any scales in the instrument were subjected to Cronbach Alpha [20] to check reliability, with an alpha of at least 0.70 considered reliable. Misunderstanding the questions was minimised by collecting data using face to face interviews though interviewer bias cannot be excluded. Individual interviews and document review ensured trustworthiness of the data through triangulation of data collection methods.

Data were entered in STATA version 16, coded and cleaned before analysis. Descriptive analysis was done to explore distribution of variables as well as identifying the common factors among the responses. Frequencies were used to describe and summarise categorical data. The study also tested associations between and among variables. Tests for association were conducted using the chi-square and interquartile ranges. Furthermore, inferential statistics in the form of ordered logistic regression was used to test the study hypothesis. Thematic analysis was done for qualitative sections of the patients’ data following the process of unitising and categorising.

### Ethical considerations

Study approval was obtained from Medical Research Council of Zimbabwe **(MRCZ A2505)** and University of KwaZulu-Natal Biomedical Research Ethics Committee **(BREC 05819)**. Permission was also obtained from Parirenyatwa hospital authorities. Participation in the study was voluntary. Written informed consent was obtained from each participant after a briefing of the study prior participating in the study. Only patients of at least 18 years and above were included. Participants were assured that all information and discussions were to remain confidential. They were informed that they may refuse to participate and that they may withdraw from the study at any time. They were also told that their decision to participate was voluntary and that any objection to participate was not going to affect any of their rights to health benefits.

## Results

A total of 408 (out of 420 distributed) patients were successfully enrolled in the study between October 2019 and September 2020, which is a 97% response rate. Table 1 is showing the socio-demographics of the CC patients. Median age (age was collected in actual years) of patients was 53 years, youngest at 28 years and oldest being 78 years old. A wide range by any standard, however it is considered strength of the study as effort was to understand CC management among all patients that zooming onto certain age category, the researchers therefore let the study lead in terms of who is available to respond and can inform the assessment. Of the respondents 313 (87%) were unemployed, 232 (57%) were married, and 43 (10.6%) had tertiary education as highest level of education. A total of 226 (55%) of the patients were from the rural areas. Majority, 402 (98.5%) of respondents were Christians, with only 6 (2%) practicing African traditional religion.

**Table 1:** Characteristics of the study population (N = 408).

Characteristics	N (%)
**Age group**	
18–35	23 (5.7)
36–49	156 (38.3)
50–64	139 (34.2)
≥65	89 (21.8)
Missing	1*
**Employment**	
Yes	90 (12.6)
No	313 (87.4)
Missing	5*
**Marital Status**	
Married	232 (56.9)
Divorced	47 (13.2)
Widowed	128 (32.5)
Never married	1 (0.5)
**Education**	
Primary	158 (38.8)
Secondary	206 (50.6)
Tertiary	43 (10.6)
Missing	1*
**Residence**	
Urban	182 (44.6)
Rural	226 (55.4)
**Religion**	
Christian	402 (98.5)
Traditional	6 (1.5)
**Co morbidity (any of HIV, Hypertension)**	
Yes	344 (84.3)
No	64 (15.7)
**HIV Status**	
Negative	130 (31.9)
Positive	278 (68.1)
**Contraception use**	
Yes	387 (95.3)
No	19 (4.7)
Missing	2*
**Parity**	
1–4	185 (45.3)
5–8	143 (35.0)
9–12	57 (14.0)
13–14	17 (4.2)
Missing	6*

*Missing count. All missing observations were not included in analysis, and no frequency is computed

Patients with co-morbidities were 344 (84%), with 278 (81%) reporting that they were living with HIV (status reported by patients). On the other hand, 387 (95%) had used contraception at some point in their lifetime.

As reported in Table 2, only 87 (21.1%) had been screened for CC before. Of the 87 that had been screened, majority, 84 (96.5%) used the VIAC method. To manage CC, majority 350 (86%) used radiotherapy, while 155 (38.2%) used chemotherapy and only 2(0.5%) used surgery. From these methods of managing CC, 370 (90.8%) reported encountering challenges like availability, affordability or machine breakdown. On the same note, 280 (69.7%) encounter challenges with pain management. Parity averages (median) 6, with minimum of 2 and maximum of 14. Treatment started 3 months (median) after diagnosis with some having to wait up to two years.

**Table 2:** CC management related characteristics (N = 408).

**Have you been vaccinated for CC**	
Yes	1 (0.3)
No	404 (99.07)
Missing	3*
**Have you been screened for CC?**	
Yes	87 (21.1)
No	321 (78.9)
**Methods of screening if Screened**	
VIAC	83 (96.5)
PAP smear	3 (3.5)
**Staging**	
1ab	1 (0.3)
2a	81 (20.0)
2b	133 (32.8)
3abc	86 (21.2)
4ab	105 (25.9)
Missing	2*
**Cancer treatment methods** ^#^	
Surgery	2 (0.5)
Radiotherapy	350 (86.0)
Chemotherapy	155 (38.2)
Missing	1*
**Have you encountered any problem/challenge with treatment methods**	
Yes	345 (90.8)
No	35 (9.2)
Missing	28*
**Have you encounter any problem/ challenge with pain management**	
Yes	280 (69.7)
No	122 (30.3)
Missing	6*
**Aspects of pharmacoepidemiology**:	
**Ever had your medicines changed**	
Yes	56 (13.8)
No	349 (86.2)
Missing	3*
**Medication giving you any problems**	
Yes	369 (93.2)
No	27 (6.8)
Missing	12*
**Experiencing any cognitive problems**	
Yes	368 (90.6)
No	38(9.4)
Missing	2*
**Ever stopped/failed taking any of the CC medication**	.
Yes	353(87.4)
No	51(12.6)
Missing	4*
**Reasons why not taking your medicines** ^#^	
Lack of money to purchase the medicines	330 (89.2)
Unavailability of the medicines	264 (71.4)
Complexity of the therapeutic regimens	100 (27.0)
Side effects	28 (7.6)
Frustration	17 (4.6)

*Missing count. All missing observations were not included in analysis, and no frequency is computed

^#^Multiple response variables- total N exceeds n

The median period since diagnosis to date of survey was 13.7 months; with one patient up to 112 months (9 years and 4 months). On average (median) the patients travelled 113km, with some travelling as far as 526 km to seek treatment. Key equipment (one radiotherapy machine) is in the capital city Harare, thereby demanding patients to travel long distances.

### Patterns and characteristics of cervical cancer management in Zimbabwe

#### Screening, diagnosis and treatment

Overall, screening was low (21.1%); however it was even very low, to none existent in those who have had many children- only 3 (3.6%) out of 83 who have been screened are in the category of highest number of children (category 4, which is 13–14 births). The association between parity and screening is statistically significant with Pearson Chi-square of 41.08 (p-value of< 0.001). It would be expected that females who have given birth would likely screen; we assess this using chi-square test (see S1 Table). The results are contrary to this proposition as women with at least one para, have mainly responded ‘No’ to the question of whether they have been screened or not.

On average (median) the patients had CC diagnosed at between stage 2b and 3abc. Based on results in S2 Table, 326 (80%) of patients cumulatively, had CC diagnosed late from stage 2b to 4ab; with only 1 (0.3%) diagnosed at initial stage (1a) and 81 (20%) diagnosed at stage 2a. Further analysis on what causes late presentation is explored and results presented later in this paper.

#### Drug utilisation and pharmacoepidemiology

Majority of the patients have been using the same chemotherapy drugs to complete their cycle, reported by 349 (86.2%), with only 56 (13.8%) having had their chemotherapy changed mostly because they would have failed to get money to buy and the drug was not available. Majority 369 (90.4%) report that the drugs were giving them problems. One of the major problems confirmed in this study is that of cognitive in nature (psychological effects), with 370 (90.6%) patients reporting it.

It is worrying that there is low adherence, with only 51(12.62%) of the patients having not stopped/failed to use the prescribed medication. It is imperative however to note that this does not reflect voluntary stoppage of chemotherapy, but also include failure to access the medication. The latter argument is revealed in the follow up question, where the greater number of the patients 330 (89.2%) indicated that not taking medicine was due to lack of money. This is followed by the next group which failed to take medicines because it was not available 264 (71.4%). On the other hand, the third popular reason for non-adherence is complexity of regimens100 (27.0%) followed by side effects as reason 28 (7.6%) and lastly frustration from not getting expected results from treatment 17 (4.6%). The patients appear to be emotionally strong as such frustration is the least cause of non-adherence as majority are religious.

In qualitative form, some of the responses above were followed up and the data was unitised and coded. S3 Table presents the results showing frequency of the emerging categories/codes in drug utilisation. The main side effects have been reported as vomiting, nausea, loss of appetite, anaemia, and peripheral neuropathy, all with above 100(24.5) patients reporting. On the other hand, morphine is the popular pain management drug reported by 257(63%) patients followed by paracetamol in 144(35.3) patients. The challenges reported are generally different forms of pain, from general body pain to pains on specific body sections/ parts. Family members, mainly spouse (husband) have been reported to be the main helper in dealing with CC among the patients. On the other hand, the main chemotherapy drug in managing CC was cisplatin identified by 285(69.9%) patients and paclitaxel by 182(44.6%) patients, and a host other supportive medicine. It can be deduced then that these drugs are also giving the patients problems due to side effects- unfortunately no further data was collected in this regard. Overall CC management depends on the Knowledge, Attitude Practice (KAP) of the patients, and this study explores this.

#### Knowledge, Attitude and Practice (KAP) among CC patients

CC patients presented with different problems like vaginal bleeding were 118 (28.9%), heavy foul discharge, 106 (26%), lower abdominal pain, 99(24.3%), side abdominal pain, 88 (21.6%), renal failure, 73(17.9%), oedema 67(16.4%) and vaginal wounds, 55 (13.5%). The top reported cause for CC was erroneously vaginal douching by149 (36.5%), other causes included were: multiple sexual partners, 147 (36.0%), unprotected sex, 87(21.3%), STIs 52 (12.7%) and HIV 51(12.5%). The real cause of CC is HPV which was mentioned by 52 (12.7%) patients only. On the other hand, patients consider diet (of one form or the other) as key to managing CC, ranging from low fat, no sugar foods; to types of foods such as black jack (herb), traditional food, and dried vegetables.

Regarding attitude (A), highest number188 (46.2%) of patients feared the cost of treatment when they were diagnosed with CC, while142 (34.8%) feared death. Patients were also afraid of side effects of treatment 137 (33.6%). On the other hand, 377 (92.5%) strongly believe that religion helps in managing CC. Only18 (4.4%) thinks that any female can get CC, while 17 (4.2%) don’t know if any female can get CC, while the majority 373 (91.4%) believe specific females, either in terms of age, or sexual behaviour are the only ones who can get CC.

In terms of practice (P), 357 (87.5%) of patients did something in addition to medical methods to manage CC. The majority of patients, 235(58%,) take some herbal medicines. In the same fashion, majority 228(58.2%) have consulted a prophet and/or traditional healer; however only 47 (11.2%) saw some changes on their conditions after consulting these religious or traditional healers. Overall, traditional healers were the most consulted, as reported by 200 (87.3%). Majority of the patients 372 (92.5%) strongly agree that religion was helping them in managing the disease. S4 Table has the details.

#### Services and medicines accessibility

Patients said that CC management is hindered by high costs; and many patients indicated that their greatest worry after diagnosis was about where to get funds to access treatment. On average (median) patients paid USD 1,620.93; the scarcity of medicine and shortage of equipment was due to the high costs. Some have paid as much as USD3, 000 per complete cycle treatment.

S5 Table presents results from questions on access to different forms of treatment, 253(67.3%) of the sample, had had problems with radiotherapy. Radiotherapy was missed by majority272 (72.7%), which was mainly due to equipment breakdown. Majority 344(85.2%) failed to purchase medicines and 370(91.4%) failed to get medicines because they were not available. Majority of the patients389 (96.1%) used cash to pay for their medical services and 384(94.0) said they get their medicines from private pharmacies where the medicines were very expensive; with medical insurance coverage being as low as 35(8.6%). Patients suggested that the government should avail the chemotherapy at public hospital pharmacies where they can buy at a reasonable price than private pharmacies where a complete cycle of chemotherapy costs USD350.

When asked what needs to be done to improve CC management patients indicated the need for subsidization (funding support) and for reliable equipment/infrastructure. Donations have been called for by most patients. In addition, awareness campaigns have been raised as one of the key considerations, where girls are also targeted with information. Vaccination programmes which are free and targeting girls and accessible to those in remote areas have also been indicated. Regular screening and practicing safe sex are some of the considerations that were put forward.

Multiple logistic regression analysis was conducted to determine the factors that explain screening behavior of patients. The results are presented in Table 3 below, showing that only employment status, level of education and co morbidities have a statistically significant effect on screening behavior (whether to screen– 1 vs. no screening done- 0). Those not employed are less likely to have been screened before compared to those in employment.

**Table 3:** Ordered logistic regression analysis for factors explaining prevention (screening uptake) (n = 408).

Factors	Odds ratio (95% CI)	p-value
Age	1.02(0.97,1.06)	0.441
Employment		
No	0.50 (0.24, 1.05)	0.066
Marriage		
Divorced	1.90(0.77, 4.70)	0.165
Widowed	0.67 (0.21, 2.10)	0.489
Never married	2365753 (0, undefined)	0.977
Education		
Secondary	9.50(2.35, 38.39)	0.002
Tertiary	59.38(11.94, 295.38)	p<0.001
Distance	1.00 (1.00, 1.01)	0.906
Location		
Rural	0.69 (0.30,1.57)	0.373
Co-morbidity		
No	0.26 (0.06,1.24)	0.091
HIV_status		
Positive	0.44 (0.12, 1.61)	0.216
Parity	0.90 (0.74,1.09)	0.294
Contraception use		
No	1.13 (0.15, 8.54)	0.907

As education level increases, one is more likely to have been screened- as can be seen by positive coefficients, meaning more likely compared to the comparator group of primary education. In addition, a higher magnitude (larger coefficient) at tertiary level is reported, meaning those with tertiary education are more likely to have been screened compared to those with secondary education as the highest level. There was an association between education and screening status (p < 0.001) after adjusting for other study variables. The odds of screening uptake among those unemployed were 9.5 times (p = 0.002) the odds of screening among those with primary education. Those with no other medical conditions are less likely to have been screened compared to those with other medical condition.

Marginal Effects for the statistically significant variables were computed to show what the probability of being screened is. S6 Table presents the results. Probability ranges from 0 to 1, with one being certain, and 0 no chance of effect. Results show that being in employment, one is 0.27 (27%) likely to have been screened compared to only 0.18 for those not in employment. Patients with tertiary level as highest level of education have a high probability of screening at 0.63; followed by those with secondary education at 0.22 and least is primary education holders at only 4% (0.04). On the other hand, patients with other conditions have highest probability of screening (0.22) compared to those without (0.14).

On the other hand, factors influencing late presentation (2b, 3abc and 4ab) have been explored using ordered logistic regression, given that the stages of late presentation 2b to 4ab are in ordinal nature. The results are presented in Table 4 below.

**Table 4:** Ordered logistic regression- factors explaining diagnosis (late presentation) (n = 408).

	Odds ratio (95% CI)	p-value
Age	1.01 (0.98, 1.04)	0.412
Employment		
Yes	Ref	
No	1.45(0.83, 2.54)	0.190
Marriage		
Married	Ref	
Divorced	2.87 (1.55, 5.31)	0.001
Widowed	2.00 (1.11, 3.59)	0.021
Never married	2.41e-06 (0, undefined)	0.987
Education		
Primary	Ref	
Secondary	0.59 (0.33, 1.05)	0.074
Tertiary	0.39 (0.17, 0.93)	0.034
Distance	1.01 (0.10, 1.00)	0.088
Location		
Urban	Ref	
Rural	0.61 (0.38, 0.99)	0.049
Co-morbidity		
Yes	Ref	
No	0.85 (0.39, 1.83)	0.670
HIV_status		
Negative	Ref	
Positive	0.70 (0.34, 1.45)	0.332
Parity	1.29 (1.16, 1.45)	<0.001
Contraception use		
Yes	Ref	
No	1.61 (0.54, 4.80)	0.398

Marriage, education, location and parity affect presentation at 5% significant level. The divorced (2.866 odds ratio (OR); p-value = 0.001) and widowed (1.997 OR, p-value = 0.021) are more likely to have late presentation than the married patients. On the other hand, as education level moves to higher levels as secondary and tertiary, one is less likely to present late (.393 OR p = .034). Facilities are overwhelmed with a lengthy waiting as machines breakdown and the waiting list is longer as reported by patients. On the other hand, regarding parity, those who have had many births are more likely to have late presentation (1.294OR p<0.001); in line with Pearson Chi-square results presented in S7 Table.

Considering association between presentation and screening, the results show that of those who have screened (87), cumulatively 72 (44.7%) have presented at least by stage 2a; and the remainder 55.3% have presented late (from stage 2b = 40%; stage 3 = 11.8% and stage 4 = 3.5%). On the other hand, of those who had not been screened (321), only 13.7% presented at stage 2a, the rest (86.3%) presented late (30.8% at stage 2b; 23.7% at stage 3, and 31.8% at stage 4). Comparing the two shows that the non-screened tend to present late, an association therefore exists, which is also confirmed by the formal test (Chi-square = 60.29; p-value < 0.001).

Eighty one percent (330) of the patients were HIV positive and Logistic regression was used to analyse this dependent variable, results are shown in Table 5.

**Table 5:** Logistic regression: Factors related to HIV status.

HIV	Odds ratio (95% CI)	p-value
Screened		
No	Ref	
Yes	3.88(0.94, 15.93)	0.060
Age		
35 years and below	Ref	
36–49 years	12.67 (2.11, 76.14)	0.006
50–64 years	2.17 (0.44, 10.65)	0.341
65 years and above	0.65(0.09, 4.90)	0.673
Employment		
Yes	Ref	
No	1.71(0.41, 7.03)	0.460
Marriage		
married	Ref	
divorced	0.54 (0.15, 1.95)	0.347
Widowed	1.85 (0.53, 6.44)	0.334
Never married	1.38e+07 (0, undefined)	0.989
Education		
Primary	Ref	
Secondary	4.98 (1.39, 17.80)	0.013
Tertiary	2.80 (0.31, 25.08)	0.358
Location		
Urban	Ref	
Rural	1.54 (0.61, 3.90)	0.360
Co-morbidity		
No	Ref	
Yes	893.96 (129.61, 6165.81)	<0.001
Parity	0.82 (0.67, 1.00)	0.054
Contraception use		
Yes	Ref	
No	0.41 (.045, 3.78)	0.432
Presentation		
1	Ref	
2	0.68 (0.18, 2.62)	0.577
3	0.45 (0.10, 1.92)	0.279
4	0.43 (0.09, 1.97)	0.278

The results show that (there is need to interpret the results with caution of not suggesting causation): Those who have screened are 3.88 times more likely to be HIV positive. This does not entail causation, as the other results show that those who get screened are more likely to be having other conditions (which maybe HIV). The screening is often aided by one visiting health facility for something else, then a suggestion (education/ awareness) of the need for CC screening is likely to come up and individual get screened. The middle aged- 36-49year olds are 12.67 times more likely to be HIV positive than the younger generation (under 35). This is the generation that was teenagers when the HIV scourge reached peak between late 80’s and early 90’s. Those with secondary education are 4.98 times more likely to be HIV positive than those with primary education as highest level. Those with some pre-existing disease/ conditions are 894 times more likely to be HIV positive. This is so because majority (81%) of the respondents indicated that they were HIV positive. The higher the parity, the less likely one is HIV positive (0.822 times < 1). This may confirm that those who are HIV positive are less likely to have many children either out of fear of transmission to partner or child at birth. Again, it is not suggesting causation as having more children does not reduce the risk of HIV contraction. Data used in this study is available in S2 File.

## Discussion

This mixed-methods study that was conducted at a central hospital in Zimbabwe found that prevention for CC was low evidenced by low screening rate due to unavailability of free screening services and knowledge about CC. Implying that majority of those that have been diagnosed of CC, had not made efforts to prevent. Most patients were diagnosed with advanced stage of the disease and belonged to low social class; unemployed, no tertiary education and no medical insurance. Socio-economic factors have been found to be associated with CC [21]. There was a lot of treatment interruption on radiotherapy due to machine breakdown as well as financial challenges. Most patients could not afford chemotherapy which was only available at private pharmacies. Most of the patients were HIV positive and were multipara. QOL was poor evidence by a lot of side effects from radiotherapy and chemotherapy and survival was low. Most patients reported to have used herbal medicines prior seeking convectional medical care. Screening was associated with being employed, education level and co-morbidities. Late stage presentation was associated with being divorced, low level of education, living in rural areas and high parity. Being HIV positive was associated with screening, low parity, and co-morbidities.

Sasagawa et al [22] reported that relative risk of CC among women with five or more births is high. There is an opportunity to increase screening, when screening is advised/recommended and embedded to postpartum clinics as suggested by literature [23]. This can further be aided by making the postpartum follow ups to be earlier, say 2–3 weeks through transformation of the health sector. Multiparity is a CC risk factor. The limited screening signifies the limited knowledge about CC within the population, to which the patients concluded that awareness campaigns are critical to win the fight against CC.

The knowledge about CC was very low with only 13% being able to say that CC is caused by HPV, which actually accounts for 99% of all CC [24]. The availability of HPV vaccine presents an opportunity to reduce morbidity and mortality of CC. HPV vaccine has become part of CC prevention in HICs [25]. The screening rate in this study of 21% was quite low due to the fact that there is no organised national screening programme as well as lack of knowledge and awareness about the diseases and its prevention. Coverage of CC screening in LMICs is averagely 19% [26]. This figure is almost the same with what was found in a study which was done in Ethiopia where screening uptake was 25% [27]. This is computed by the fact that majority of the patients are in rural areas and are known to have low uptake of CC screening [28]. There is need to establish population-based CC screening. Cost of screening was seen as a barrier to screening so there is need to subsidise the cost of screening like in private where VIAC costs USD20 and Pap smear costs USD60.

Cervical cancer survivors can be included in awareness campaigns as knowing a person with CC was associated with knowledge about CC and high screening uptake [29]. Knowledge about CC was elementary and there was high prevalence of ignorance about causes of CC where 36% of the patients thought that CC was caused by vaginal douching. There is need to step up health education and campaigns. Globally effective implementation of screening programs based on cytological analysis, helped to reduce CC [30]. Religion is known to influence knowledge and screening practices [31], therefore understanding the nature of beliefs within each religion is of importance. Given that majority of patients pay the bills on their own, lack of employment means lack of funding, hence failure to screen is due to lack of funding as reported in [32]. The result on education is supported by the works of Fentie et al [33] asserting that knowledge is a powerful predictor, and so is marital status [34]. Education relates to literacy and therefore awareness is likely to be high; with an added advantage of positive attitudes as knowledge increases.

Those with no other medical conditions were less likely to have been screened compared to those with other medical condition. This may be due to the fact that patients with other conditions may have been recommended for screening while going for other routine check-ups- as there is more interaction with medical practitioners and facilities. This can be interpreted in the same way as in the study done by Nwabichie et al [34] who found that having a regular health care provider increases screening uptake. Also given the distance travelled by some patients, having another condition brings one closer to the relevant facilities, and then opportunities to be screened. Screening is very low which is also attributable to poor socio-economic profiles of households in Sub-Saharan Africa (Zimbabwe included), the costs, and unavailability of the screening service. CC can be prevented if timely screening takes place [35], however the uptake of the screening process is unfortunately not yet at acceptable levels.

In this study there were two CC intervals, that is time from diagnosis to commencement of treatment which was a median of 3 months and time the patient had lived since diagnosis of CC was a median of 14 months. Model of Pathways to Treatment explain CC journey starting from symptom recognition to help seeking, diagnosis and then treatment. It is as an iterative process with events and processes with distinguishable intervals. These events and intervals are influenced by factors such as patient demographics, healthcare access, disease factors like rate of progression and histological subtypes [36]. Policymakers can gain insight into actual points along the journey where delay can occur and hence provide opportunities to design targeted interventions. There is need to target patients and clinicians in public awareness campaigns and continuous professional development.

Fifty-eight percent admitted to have used herbs before coming for conventional medical care and admitted not to have seen any change but their disease was worsening or delayed them in seeking convectional medical care. In a study which was done in Kenya, 38% reported use of traditional herbs but there was no difference in treatment outcomes between those who used and those who did not [37]. Traditional healers can be offered basic training to alert them to know symptoms of CC and refer these patients to health centres.

Majority of patients in this study were HIV positive (81%), higher than what was found in a study which was done in Botswana where 65% of the CC patients were HIV positive [29]. In Kenya the incidence of CC was 900 per 100 000 in women with HIV- AIDS but 10 per 100 000 in the general population [37]. Cervical cancer is an AIDS defining condition and anti-retroviral therapy prolongs survival among HIV positive but do not prevent them from developing CC [38]. In Ukraine HIV infected women receive CC screening as part of HIV care [39] and routine reminders as well as appointments for screening instead of relying on the patients’ initiative as HIV positive women are supposed to be screened yearly [40] since they have a 4 fold excess risk of developing CC because of reduced immunity and HIV positive women can develop CC 10 years before those who are HIV negative [41]. In Zimbabwe CC is the most commonly diagnosed cancer among women and its high incidence is in correlation with the high HIV prevalence in the country [42].

Less than 1% (1 patient) had surgery as a form of CC treatment option in this study because most CC patients present at late stage precluding the use of surgery. Eighty percent presented with advanced stage exactly the same percentage that was found in a study by Ndlovu and Kambarami [43], a more intensified screening programme is necessary. Patients require standard curative treatment with external beam radiation and brachytherapy with or without concomitant chemotherapy. Institutional delay and patient delay result in advanced stage at diagnosis. Treatment from traditional healers and herbalist resulted in delay in seeking health care and in this study 58% of the patient said they had consulted a traditional healer/prophet before coming to the hospital. Women cannot be solely be responsible for late presentation because lack of suspicion of CC by health care professionals and the lack of prioritisation of CC management by Health Departments were major contributors to presentation of advance stage at diagnosis in a study which was done in South Africa [44]. Late presentation is a problem in fighting CC as it increases mortality rate as has been found in some CC studies [45]. The major factor as reported in literature is poor socioeconomic factors [46]; to which Zimbabwe depict the extreme of such deprivation.

Early detection of CC is important due to the relationship between stage at diagnosis and survival in this study the average time the patients had lived after diagnosed with CC was 14 months. This result is almost the same with that which was found in Kenya where the mean time to death after the onset of treatment was 15.1 months while the median survival was 15.0 months [37]. Five-year survival rate is 91% for localised CC at diagnosis which falls to 57% for distant stage CC [47]. CC requires continuum of care as well as comprehensive programmes which is dependent on the stage of the CC to improve health outcomes.

CC management is not just working; passion for the patient is needed as patients narrated their CC journey and the challenges they were facing like need for sanitary pads due to heavy bleeding and the abnormal foul discharge that nobody enjoy being close to them let alone the challenge of accommodation as they come for chemo radiotherapy since most of the patients were coming from all over the country. Patients perceive diagnosis with CC as a death sentence as evidenced by 30% saying after diagnosed of CC they feared death and 47% cited fear of treatment costs. Honouring and commitment to the Abuja declaration which says at least 15% of national budget must go to health [48] will solve some of these health challenges. The fact that CC is a disease of the poor demonstrating inequities of access to health care [49] was also supported in this study where only 13% were employed,8% had medical insurance and 10% had tertiary education. According to Tapera et al only 10% of the Zimbabwean population were on medical aid [42]. literature indicating that non-adherence in Africa is often due to non-availability and costs of drugs [32].

Most of the patients resided in rural areas 55% just like in a study which was done in Ethiopia where 57% resided in rural areas [50]. It is a fact that five out of six women with CC live in developing countries, which possess only 5% of the global resources for cancer control [37]. There is need for health care facilities to develop mechanisms for financial support for CC patients. Financial challenges included money for diagnosis, treatment, and travel and hygiene maintenance. Governments must increase budgetary allocation for CC management and find ways of subsidising CC management for most patients to afford.

Concomitant chemo radiotherapy using cisplatin as the chemotherapeutic radio sensitising agent is the standard of care for treatment of CC in SSA [51] which was exactly what was found in this study. Ninety- one percent failed to purchase their chemotherapy because it was not available, at private pharmacies the whole cycle would need USD350 and 85% failed to purchase chemotherapy because of financial constraints, corroborating [52]. Due to limited availability and financial limitations chemotherapy was not administered on regular basis and only 38% used chemotherapy. Low adherence is one of the significant problems in managing CC [32]. Chemotherapy drug shortages are occurring at an alarming rate worldwide due to a lack of financial incentives for manufacturing companies due to inexpensive injectables and oral generic forms of chemotherapy drugs [53].

Human cost of stock outs of chemotherapy drugs is significant, because efficacy depends on delivering the full dose on schedule for a specific number of treatment cycles, treatment interruption reduce the potential long-term remission rate as well as curative potential to zero and that remission might not be achieved when medication is reinstituted. This explains the low survival of a mean of 14 months from date of diagnosis. Five year survival rate was 19% for Zimbabwe, 22% for Gambia and13% for Uganda [50]. Lack of early detection activities and therapeutic options can explain the lower survival rates. Side effects in treated participants were exactly as those documented in literature which included neurological, hematologic, and renal toxicities [51].

The unavailability of radiotherapy was pitiable as 73% of respondents have missed a cession of radiotherapy mainly because the machine had broken down at public hospital, while at private facility the costs were prohibitively high, with a complete cycle for external beam costing USD3, 500. The country has two machines in public sector, and is not uncommon in Africa as Ethiopia also had one radiotherapy for the whole country [54]. Even in developed countries like US inaccessibility of health facilities is present, with more than 2 million CC survivors not getting one or more needed medical services because of financial challenges among the Hispanic and black survivors [55]. All these factors lead to treatment interruption, which is the failure to execute treatment plan of a patient [56]. Radiotherapy centres should strive to acquire at least two radiotherapy machines to have back-up. Radiation oncology facility needs to have policies for treatment interruption management and documentation to achieve better radiotherapy care. In addition, given late presentation at diagnosis, a small proportion of women with CC would benefit from chemo radiotherapy. In a study done by Akinlade et al [56], most of the patients depended on their relatives, church members and their children for financial assistance; most of whom did not have any formal employment or steady source of income, which is exactly in line with what we found out in this study.

Quality of life (QOL) of patients in this study was quite compromised, with 90% having cognitive problems, 93% experienced side effects with chemotherapy, 70% pain was not under control and 67% reported challenges with radiotherapy. QOL constitutes a state of well-being which includes physical, psychological and social well-being and patient’s satisfaction with level of functioning, control of disease and treatment symptoms. In a study which was done in Kenya, they reported same low QOL with the following functions affected; physical 79%, role 75%,cognitive 56%,social 71% and financial aspects 63% [57]. Almost all the patients who undergo radiotherapy experienced some side effects which were exactly those found in a study which was done in Ethiopia [50], the side effects of radiotherapy included radiation proctitis, subcutaneous fibrosis of the suprapubic tissue and vaginal strictures, radiotherapy dermatitis, diarrhoea and incontinent because of vesicovaginal fistula. In literature, Sun et al [58], reported that multiple demographic and clinical factors explain side effects and their severity.

Diagnosis and treatment of CC requires a health system which is functional, interactive as well as responsive to be able to identify, diagnose and treat the diseases. In terms of challenges at presentation and pain management the result in this study is almost the same with what was found by Moelle et al where at the time of presentation, 90% of all patients suffered from abdominal pain, which was severe and morphine injections were given to only 14% of all patients, and tramadol was administered to 43% of all patients, 4% received paracetamol and 27% did not receive any analgesic [54]. Awareness campaigns and CC education should target the Early Middle Age and those at their secondary educational level since the relationship with the HIV positive status was significant. In addition, educational campaigns should also reach males, as the study showed that some males (sons, brothers, and spouses) were the available support for CC patients.

The strength of the study is in its ability to implement a mixed method approach and face to face interviews using a semi- structured questionnaire resulting in a vivid picture of CC management status in Zimbabwe. The fact that all departments that deal with CC patients at the hospital were included in the study; ensured robustness of the study findings. Like in any interviewer administered questionnaire study, social desirability bias is a limiting factor. Some data relied on self-reporting from patients which might have been subjected to recall bias effect which are inherent in-patient interview studies. The study was hospital-based involving women who had reached the hospital; characteristics of women who have CC but who did not reach the study hospital remain unknown and could be facing worse challenges in CC management. Interviewer bias cannot be completely excluded. The study excluded women who received CC care from private health institutions. Further studies can be done to get health professionals’ perspective on CC management as they are involved in the management.

## Conclusions

Capacity building in gynaecologic, radiotherapy, oncology, chemotherapy and palliative care services is critical. Women at risk of developing CC need accurate information to understand prevention methods and to use screening services. Knowledge about CC was low hence awareness campaigns and education should be done, access and availability of CC screening is essential to improve CC screening uptake. Education campaigns must focus on improving attitudes, increasing risk perceptions and encouraging screening when free from signs and symptoms and CC screening should form part of discussion between health professionals and women when they seek health care. Radiotherapy centres should strive to acquire at least two radiotherapy machines. Economic challenges have affected the capacity of the health system to provide comprehensive care for CC patients hence a strong political will and mobilisation of resources to build capacity for preventing and treatment of CC. Majority of the patients belonged to the low socio-economic status class. Interruption and absence of radiotherapy limited the probability of curative treatment. Knowledge about CC was poor and CC screening rate was low, these findings underscore the need to increase health education on CC prevention and early detection among HIV-positive women and the general populace. Quantification of chemotherapy can be done using resource-sensitive treatment strategy can help reduce stock- outs and produce an effective and efficient procurement systems. National screening programmes and the provision of radiotherapy services are major priorities needed. There was severe deterioration in QOL as a result of diagnosis and therapies hence the need for management services that can ensure coping with CC for patients. Advance stage at presentation is the main prognostic factor for low survival. Chemotherapy and analgesics must be affordable and available. Health system is constrained to provide CC prevention, treatment and palliative care.

## Supporting information

S1 File Research instrument. (DOCX)

S2 File Survey data. (XLSX)

S1 Table Chi-square test of association between multiparty and CC screening. (DOCX)

S2 Table Descriptive statistics of presentation stage. (DOCX)

S3 Table Drug utilisation. (DOCX)

S4 Table Attitude/Practice towards CC management. (DOCX)

S5 Table Treatment accessibility and availability. (DOCX)

S6 Table Marginal effects for regression results for screening. (DOCX)

S7 Table Association between presentation stage and CC screening. (DOCX)
