# Supplementary material for: Cervical cancer management in Zimbabwe (2019–2020)
Source: PLoS One. 2022 Sep 21;17(9):e0274884. doi: 10.1371/journal.pone.0274884 (PMC9491541; doi:10.1371/journal.pone.0274884)
Supplement: S1 File — (DOCX) [file pone.0274884.s001.docx]

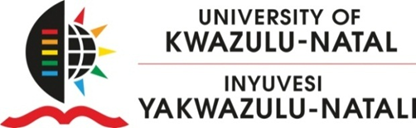


**Study title: SITUATIONAL ANALYSIS OF CERVICAL CANCER MANAGEMENT IN ZIMBABWE**

**BREC Approval No.:05819**

**MRCZ Approval No:A2505**

**Questionnaire No.**  [ ][ ][ ]

**Study site ID number:** [ ][ ][ ] **Date:** [ ][ ] / [ ][ ] / [ ][ ][ ][ ]

dd mm yyyy

**Instructions**

1. **All participants should be cervical cancer patients not critically ill or bedridden**
2. **This questionnaire is pre-coded, please mark clear with cross (X) in the space provided.**

**A. SOCIO-DEMOGRAPHICS**

1. Age
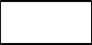
 Employed Yes
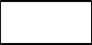
 No
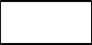
 Occupation....................................

Marital Status Married
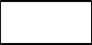
Single
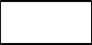


Educational Level: Primary
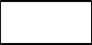
 Secondary
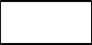
 Tertiary
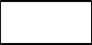


Residential Urban
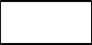
 Rural
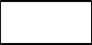


What is your religon? Christian
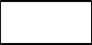
 Muslim
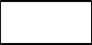
 Traditional
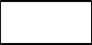
 Other.....................

Are you having any other disease/condition? Yes
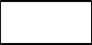
No
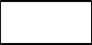
 Diseases/condition.............................

Date of diagnosis:
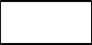
 Date Censoring
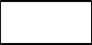
 Survival Time
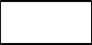


HIV Status
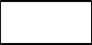


**B. SCREENING, DIAGNOSIS AND TREATMENT**

2. Were you ever vaccinated against cervical cancer? Yes
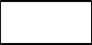
No
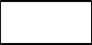


3a.Were you ever screened for cervical cancer? Yes
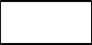
 No
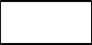


3b. what method of screening was used? ................................................................................................

4. How were you diagnosed of cervical cancer?...........................................................................................

5. After how long from diagnosis did you commence treatment?...................................................................

6. What methods have been used to manage your cancer? Surgery
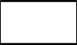
Radiotherapy
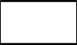
 Chemo
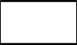


6b.Did you face any challenges with the above procedures? Yes
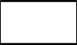
 No
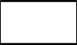


6c What were the challenges?..............................................................................................................................

7a.What are you using to manage pain.................................................................................................................

7b.Are you having challenges with pain management?......................................................................................

7c.What are the challenges?.......................................................................................................................................

**C. DRUG UTILISATION AND PHARMACOEPIDEMIOLOGY**

8. Medicines (all)

(i)…………………..……………………………………………….

(ii)………………….………………………………………………….

(iii)………………….………………………………………………….

(iv)…………………..………………………………………………….

(v)…………………..………………………………………………….

8a. Have you ever had your medicines changed? Yes
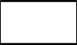
 No
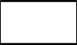


8b.What were the reasons for changing?................................................................................

8c. Is the medication you are taking giving you any problems? Yes
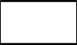
 No
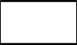


8d. What were the problems?.............................................................................................................................................

...........................................................................................................................................................................................

.........................................................................................................................................................................................

9a. Are you experiencing any cognitive problems? Yes
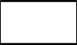
 No
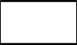


9b.What problems are you having?...................................................................................................................................

10a. Have you ever stopped taking any of the medicines or failed to take your medicines?

Yes
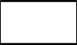
 No
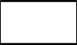


10b which of the following reasons has contributed to your not taking your medicines?

1. Frustration
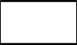


2. Side effects
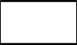


3. Complexity of the therapeutic regimens
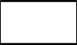


4. Lack of money to purchase the medicines
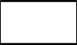


5. Unavailability of the medicines
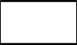


6. Other reasons ……………………………………………

**D. KNOWLEDGE, ATTITUDE AND PRACTICE**

11.May you say everything you know about cervical cancer.............................................................................

........................................................................................................................................................................

.......................................................................................................................................................................

12a. Other than taking medicines, do you do something else to control the cancer?

Yes
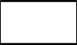
 No
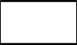


12b. What else do you do to manage the cancer? ..................................................................................................

13c. Is religion helping you in the control of cancer? Strongly agree
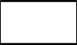
 Agree
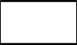
 Neutral
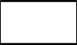
 Disagree
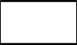
 Strongly Disagree
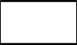


13d. How is religion helping you in the cancer management?............................................................ ................................................................................................................................................................

.................................................................................................................................................................

14a. Do you take any herbal medicines**?** Yes
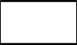
 No
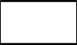
If Yes, which one.....................................

14b.Have you ever consulted a prophet or traditional healer for help? NO
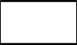
 Traditional healer
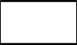
 Prophet
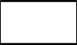


14c. How did they help you?......................................................................................................................................

14d.Did you see some changes after such consultation? Yes
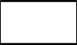
 No
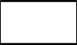
What were the changes................................

15. What worries you most after you diagnosed with cancer?

Fear of death Fear of other related diseases

Cost of treatment Side effects of medication

Other………………………………………………………………………………………………………………………………………………….

16. Where else are you getting help for your cancer management?.......................................................................

**E. SERVICES AND MEDICINES ACCESSIBILITY**

17a. Did you ever have a surgery done on you on treating cervical cancer? Yes
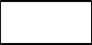
 No
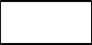


17b.Did you face any challenges? Yes
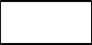
 No
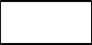


17c.What were the challenges?............................................................................................................................

17d.What do you think can be done to improve the service?..............................................................................

18a.Have you received any radiotherapy? Yes
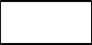
 No
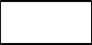


18b Did you face any challenges with the processes at radiotherapy? Yes
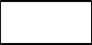
 No
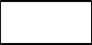


18cWhat were the challenges?.................................................................................................................

18d.What do you think can be done to avoid such challenges?........................................................................

19a.Have you ever missed your radiotherapy sessions? Yes
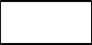
No
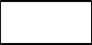


19b.What were the reasons for missing the sessions?......................................................................................

20a. How do you pay for your medical services?

Cash
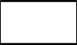
 Medical aid
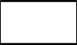
 Social welfare
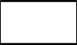
 other (please specify)……………………..

20b. If using cash how much do you spend per month on cervical cancer management? .........................

20c. Who pays for your medicines? ....................,…………….........................................................

20d. Do you sometimes fail to purchase medicines? Yes
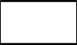
 No
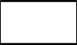
If YES state the reasons for failing to purchase medicines……………………………………………………………………

20e. What do you think should be done to improve affordability**?**

………………………………… ……………………………………………………..

21a.Do you sometimes fail to get your medicine because it will not be available?

Yes
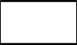
 No
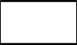


21b. Where do you often get your medicines?

Private pharmacies
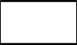
Clinics
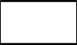
 Central hospitals
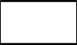
 Other (please specify)…………………..

21c. What do you think should be done to improve availability?........................................................

…………………………………………………………………………………………………………..

22. Would you like to say something about cervical cancer?..............................

........................................................................................................................................................................

**END OF QUESTIONNAIRE**

**THANK YOU**
