# Supplementary material for: Cervical cancer management in Zimbabwe (2019–2020)
Source: PLoS One. 2022 Sep 21;17(9):e0274884. doi: 10.1371/journal.pone.0274884 (PMC9491541; doi:10.1371/journal.pone.0274884)
Supplement: S1 Table — (DOCX) [file pone.0274884.s003.docx]

**S1 Table. Chi-square test of association between Multiparity and CC screening**

| MULTIGRAVIDITY | Ever been screened? | | |
| --- | --- | --- | --- |
|  | Yes | No | Total |
| 0 | 1 | 1 | 2 |
|  | 50.00 | 50.00 | 100.00 |
| 1 | 4 | 7 | 11 |
|  | 36.36 | 63.64 | 100.00 |
| 2 | 15 | 28 | 43 |
|  | 34.88 | 65.12 | 100.00 |
| 3 | 25 | 41 | 66 |
|  | 37.88 | 62.12 | 100.00 |
| 4 | 15 | 50 | 65 |
|  | 23.08 | 76.92 | 100.00 |
| 5 | 10 | 34 | 44 |
|  | 22.73 | 77.27 | 100.00 |
| 6 | 8 | 37 | 45 |
|  | 17.78 | 82.22 | 100.00 |
| 7 | 2 | 28 | 30 |
|  | 6.67 | 93.33 | 100.00 |
| 8 | 1 | 23 | 24 |
|  | 4.17 | 95.83 | 100.00 |
| 9 | 1 | 12 | 13 |
|  | 7.69 | 92.31 | 100.00 |
| 10 | 0 | 16 | 16 |
|  | 0.00 | 100.00 | 100.00 |
| 11 | 1 | 15 | 16 |
|  | 6.25 | 93.75 | 100.00 |
| 12 | 0 | 12 | 12 |
|  | 0.00 | 100.00 | 100.00 |
| 13 | 0 | 16 | 16 |
|  | 0.00 | 100.00 | 100.00 |
| 14 | 0 | 1 | 1 |
|  | 0.00 | 100.00 | 100.00 |
| Total | 83 | 321 | 404 |
|  | 20.54 | 79.46 | 100.00 |
| Pearson chi2(14) = 43.35 | | | Pr = 0.000 |

Source: Own computation based on survey data
