# Supplementary material for: Cervical cancer management in Zimbabwe (2019–2020)
Source: PLoS One. 2022 Sep 21;17(9):e0274884. doi: 10.1371/journal.pone.0274884 (PMC9491541; doi:10.1371/journal.pone.0274884)
Supplement: S2 Table — (DOCX) [file pone.0274884.s004.docx]

**S2 Table. Descriptive statistics of Presentation stage**

| Presentation stage | Freq. | Percent | Cum. |
| --- | --- | --- | --- |
| 1ab | 1 | 0.25 | 0.25 |
| 2a | 81 | 19.95 | 20.20 |
| **2b** | **133** | **32.76** | **52.96** |
| **3 abc** | **86** | **21.18** | **74.14** |
| **4 ab** | **105** | **25.86** | **100.00** |
| Total | 406 | 100.00 |  |

Source: Own computation based on survey data
