# Supplementary material for: Cervical cancer management in Zimbabwe (2019–2020)
Source: PLoS One. 2022 Sep 21;17(9):e0274884. doi: 10.1371/journal.pone.0274884 (PMC9491541; doi:10.1371/journal.pone.0274884)
Supplement: S3 Table — (DOCX) [file pone.0274884.s005.docx]

**S3 Table. Drug utilisation**

| **Challenges** | **Freq.** | **Pain management solution** | **Freq.** | **Challenges faced** | **Freq.** | **Who is helping** | **Freq.** | **Medicine type** | **Freq.** |
| --- | --- | --- | --- | --- | --- | --- | --- | --- | --- |
| vomiting | 321 | morphine | 257 | General body pain | 229 | husband | 192 | cisplatin | 285 |
| nausea | 309 | paracetamol | 144 | Backache | 119 | children | 109 | metoclopramide | 217 |
| Loss of appetite | 219 | tramadol | 59 | Vaginal bleeding | 108 | daughter | 21 | paclitaxel | 182 |
| anaemia | 164 | didofenac | 17 | Lower abdominal pains | 98 | sister | 20 | Folic acid | 176 |
| peripheral neuropathy | 138 | indomethacin | 16 | Dysuria | 53 | son | 18 | palonosetron | 165 |
| alopecia | 99 |  |  | vaginal stenosis | 43 | family | 15 | ferrous sulphate | 152 |
| Constipation | 95 |  |  | Radiation dermatitis | 43 | brother | 11 | ciprofloxacin | 109 |
| mucositis, | 93 |  |  | Vesco vaginal fistula | 25 | friend | 11 | metronidazole | 94 |
| General body weakness | 92 |  |  |  |  |  |  | ranitidine | 63 |
| renal dysfunction | 91 |  |  |  |  |  |  | promethazine | 59 |
| headache | 58 |  |  |  |  |  |  | ondansetron | 50 |
| Weight loss | 46 |  |  |  |  |  |  | tranexamic | 49 |
|  |  |  |  |  |  |  |  | dexamethasone | 46 |

Source: Own computation based on survey data
