# Supplementary material for: Cervical cancer management in Zimbabwe (2019–2020)
Source: PLoS One. 2022 Sep 21;17(9):e0274884. doi: 10.1371/journal.pone.0274884 (PMC9491541; doi:10.1371/journal.pone.0274884)
Supplement: S4 Table — (DOCX) [file pone.0274884.s006.docx]

**S4 Table. Attitude/practice towards CC management**

| Do you do something else to control the cancer? | Freq. | Percent | Cum. |
| --- | --- | --- | --- |
| Yes | **357** | **87.50** | **87.50** |
| No | **51** | **12.50** | **100.00** |
| Total | **408** | **100.00** |  |
|  |  |  |  |
| Is religion helping you | **Freq.** | **Percent** | **Cum.** |
| STRONGLY AGREE | **372** | **92.54** | **92.54** |
| AGREE | **23** | **5.72** | **98.26** |
| NEUTRAL | **4** | **1.00** | **99.25** |
| DISAGREE | **2** | **0.50** | **99.75** |
| STRONGLY DISAGREE | **1** | **0.25** | **100.00** |
| Total | **402** | **100.00** |  |
|  |  |  |  |
| Take any herbal medicines | **Freq.** | **Percent** | **Cum.** |
| Yes | **235** | **58.02** | **58.02** |
| No | **170** | **41.98** | **100.00** |
| Total | **405** | **100.00** |  |
|  |  |  |  |
| Ever consulted a prophet or traditional healer | **Freq.** | **Percent** | **Cum.** |
| Yes | **228** | **59.22** | **59.22** |
| No | **157** | **40.78** | **100.00** |
| Total | **385** | **100.00** |  |
|  |  |  |  |
| Seen some changes after consultation | **Freq.** | **Percent** | **Cum.** |
| Yes | **43** | **11.20** | **11.20** |
| No | **341** | **88.80** | **100.00** |
| Total | **384** | **100.00** |  |
|  | **Frequency** | **Percent of responses** | **Percent of cases** |
|  |  |  |  |
| Traditional healer | **200** | **71.17** | **87.34** |
| Prophet | **73** | **25.98** | **31.88** |
| Spiritual leader | **8** | **2.85** | **3.49** |
| Total | **281** | **100.00** | **122.71** |
| Worry after diagnosis with CC | **Frequency** | **Percent of responses** | **Percent of cases** |
| Fear of death | **142** | **30.28** | **69.61** |
| Cost of treatment | **190** | **40.51** | **93.14** |
| Side effects of medication | **137** | **29.21** | **67.16** |
| Total | **469** | **100.00** | **229.90** |

Source: Own computation based on survey data
