# Supplementary material for: Cervical cancer management in Zimbabwe (2019–2020)
Source: PLoS One. 2022 Sep 21;17(9):e0274884. doi: 10.1371/journal.pone.0274884 (PMC9491541; doi:10.1371/journal.pone.0274884)
Supplement: S5 Table — (DOCX) [file pone.0274884.s007.docx]

**S5 Table. Treatment accessibility and availability**

|  | Freq. | Percent | Cum. |
| --- | --- | --- | --- |
|  |  |  |  |
| Faced any challenges with radiotherapy | **Freq.** | **Percent** | **Cum.** |
| Yes | **253** | **67.29** | **67.29** |
| No | **123** | **32.71** | **100.00** |
| Total | **376** | **100.00** |  |
|  |  |  |  |
| Ever missed your radiotherapy | **Freq.** | **Percent** | **Cum.** |
| Yes | **274** | **72.68** | **72.68** |
| No | **103** | **27.32** | **100.00** |
| Total | **377** | **100.00** |  |
|  |  |  |  |
| Ever failed to purchase medicines | **Freq.** | **Percent** | **Cum.** |
| Yes | **344** | **85.15** | **85.15** |
| No | **60** | **14.85** | **100.00** |
| Total | **404** | **100.00** |  |
|  |  |  |  |
| fail to get your medicine because it will not be available | **Freq.** | **Percent** | **Cum.** |
| Yes | **370** | **91.36** | **91.61** |
| No | **35** | **8.64** | **100.00** |
| Total | **405** | **100.00** |  |
|  |  |  |  |
| How do you pay for your medical services | **Frequency** | **Percent of responses** | **Percent of cases** |
| Cash | **389** | **90.47** | **96.05** |
| Medical Insurance | **35** | **8.14** | **8.64** |
| NGO | **6** | **1.40** | **1.48** |
| Total | **430** | **100.00** | **106.17** |
|  |  |  |  |
| Where do you often get your medicines | **Frequency** | **Percent of responses** | **Percent of cases** |
| Private pharmacies | **384** | **93.71** | **174.04** |
| Clinics | **4** | **0.56** | **1.04** |
| Central hospitals | **326** | **5.59** | **10.38** |
| Other | **1** | **0.14** | **0.26** |
| Total | **715** | **100.00** | **185.71** |

Source: Own computation based on survey data
