# Supplementary material for: Cervical cancer management in Zimbabwe (2019–2020)
Source: PLoS One. 2022 Sep 21;17(9):e0274884. doi: 10.1371/journal.pone.0274884 (PMC9491541; doi:10.1371/journal.pone.0274884)
Supplement: S6 Table — (DOCX) [file pone.0274884.s008.docx]

**S6 Table. Marginal Effects for Regression results for screening**

| Predictive margins Number of obs = 398 | | | | | | |
| --- | --- | --- | --- | --- | --- | --- |
| Model VCE : OIM | | | | | | |
| Expression : Pr(Screening), predict() | | | | | | |
| Delta method | | | | | | |
|  | Margin | Std. Err. | z | P>z | [95% Conf. Interval] | |
|  |  |  |  |  |  |  |
| **EMPLOYMENT** |  |  |  |  |  |  |
| **Yes** | **.2723221** | **.0443873** | **6.14** | **0.000** | **.1853247** | **.3593196** |
| **No** | **.1838269** | **.0232845** | **7.89** | **0.000** | **.1381901** | **.2294637** |
|  |  |  |  |  |  |  |
| MARRIAGE |  |  |  |  |  |  |
| MARRIED | .1999261 | .0206843 | 9.67 | 0.000 | .1593857 | .2404666 |
| DIVORCED | .3094701 | .0585359 | 5.29 | 0.000 | .1947417 | .4241984 |
| WIDOWED | .1876598 | .0500574 | 3.75 | 0.000 | .0895491 | .2857704 |
|  |  |  |  |  |  |  |
| **EDUCATION** |  |  |  |  |  |  |
| **PRIMARY** | **.0398603** | **.0215822** | **1.85** | **0.065** | **.0024401** | **.0821607** |
| **SECONDARY** | **.2205549** | **.0333935** | **6.60** | **0.000** | **.1551048** | **.286005** |
| **TETIARY** | **.6255694** | **.0950076** | **6.58** | **0.000** | **.439358** | **.8117808** |
|  |  |  |  |  |  |  |
| RESIDENCE |  |  |  |  |  |  |
| URBAN | .223999 | .0229096 | 9.78 | 0.000 | .179097 | .268901 |
| RURAL | .1815601 | .0351859 | 5.16 | 0.000 | .1125969 | .2505233 |
|  |  |  |  |  |  |  |
| **OTHER CONDITION** |  |  |  |  |  |  |
| **Yes** | **.2225302** | **.0209828** | **10.61** | **0.000** | **.1814046** | **.2636557** |
| **No** | **.1434474** | **.0540306** | **2.65** | **0.008** | **.0375494** | **.2493454** |
|  |  |  |  |  |  |  |
| HIV_s |  |  |  |  |  |  |
| negative | .2585186 | .0652707 | 3.96 | 0.000 | .1305904 | .3864468 |
| positive | .2029022 | .0191572 | 10.59 | 0.000 | .1653548 | .2404495 |
|  |  |  |  |  |  |  |
| CONTRACEPTIONUSE |  |  |  |  |  |  |
| Yes | .2057233 | .0171396 | 12.00 | 0.000 | .1721303 | .2393163 |
| No | .3854563 | .1170835 | 3.29 | 0.001 | .1559769 | .6149358 |

Source: Own computation based on survey data
