# Supplementary material for: Cervical cancer management in Zimbabwe (2019–2020)
Source: PLoS One. 2022 Sep 21;17(9):e0274884. doi: 10.1371/journal.pone.0274884 (PMC9491541; doi:10.1371/journal.pone.0274884)
Supplement: S7 Table — (DOCX) [file pone.0274884.s009.docx]

**S7 Table. Association between presentation stage and CC screening**

| Presentation stage | | | | | | |
| --- | --- | --- | --- | --- | --- | --- |
| CC Screening | 1ab | 2a | 2b | 3abc | 4 ab | Total |
|  |  |  |  |  |  |  |
| Yes | 1 | 37 | 34 | 10 | 3 | 85 |
|  | **1.18** | **43.53** | **40.00** | **11.76** | **3.53** | **100.00** |
|  |  |  |  |  |  |  |
| No | 0 | 44 | 99 | 76 | 102 | 321 |
|  | **0.00** | **13.71** | **30.84** | **23.68** | **31.78** | **100.00** |
|  |  |  |  |  |  |  |
| Total | 1 | 81 | 133 | 86 | 105 | 406 |
|  | 0.25 | 19.95 | 32.76 | 21.18 | 25.86 | 100.00 |
|  | | | | | | |
| Pearson chi2(4) = 60.6900 Pr = 0.000 | | | | | | |
|  | | | | | | |
| Key (cells order) | | | | | | |
| frequency | | | | | | |
| row percentage | | | | | | |

Source: Own computation based on survey data
